# UAV Landing Using Computer Vision Techniques for Human Detection †

**DOI:** 10.3390/s20030613

**Published:** 2020-01-22

**Authors:** David Safadinho, João Ramos, Roberto Ribeiro, Vítor Filipe, João Barroso, António Pereira

**Affiliations:** 1 School of Technology and Management, Computer Science and Communication Research Centre, Polytechnic Institute of Leiria, Campus 2, Morro do Lena – Alto do Vieiro, Apartado 4163, 2411-901 Leiria, Portugal; davidsafadinho.12@gmail.com (D.S.); jr.joaoramos@outlook.com (J.R.); eng.rob.ribeiro@gmail.com (R.R.); 2 INESC TEC and University of Trás-os-Montes e Alto Douro, Quinta de Prados, 5001-801 Vila Real, Portugal; vfilipe@utad.pt (V.F.); jbarroso@utad.pt (J.B.); 3 INOV INESC INOVAÇÃO, Institute of New Technologies, Leiria Office, Campus 2, Morro do Lena – Alto do Vieiro, Apartado 4163, 2411-901 Leiria, Portugal

**Keywords:** autonomous delivery, computer vision, deep neural networks, intelligent vehicles, internet of things, next generation services, real-time systems, remote sensing, unmanned aerial vehicles, unmanned aircraft systems

## Abstract

The capability of drones to perform autonomous missions has led retail companies to use them for deliveries, saving time and human resources. In these services, the delivery depends on the Global Positioning System (GPS) to define an approximate landing point. However, the landscape can interfere with the satellite signal (e.g., tall buildings), reducing the accuracy of this approach. Changes in the environment can also invalidate the security of a previously defined landing site (e.g., irregular terrain, swimming pool). Therefore, the main goal of this work is to improve the process of goods delivery using drones, focusing on the detection of the potential receiver. We developed a solution that has been improved along its iterative assessment composed of five test scenarios. The built prototype complements the GPS through Computer Vision (CV) algorithms, based on Convolutional Neural Networks (CNN), running in a Raspberry Pi 3 with a Pi NoIR Camera (i.e., No InfraRed—without infrared filter). The experiments were performed with the models Single Shot Detector (SSD) MobileNet-V2, and SSDLite-MobileNet-V2. The best results were obtained in the afternoon, with the SSDLite architecture, for distances and heights between 2.5–10 m, with recalls from 59%–76%. The results confirm that a low computing power and cost-effective system can perform aerial human detection, estimating the landing position without an additional visual marker.

## 1. Introduction

In developed countries there is a solid delivery network assured by specialized companies. However, the volume of products to deliver is continuously increasing due to the growth of online shopping, which leads to the inclusion of autonomous and intelligent systems to speed up the flow of the packages. The evolution of Unmanned Aerial Vehicles (UAV), especially vertical take-off drones, has been increasing the potential of Unmanned Airborne Systems (UAS), which creates the opportunity to fulfil this need for delivering supplies in a fast fashion [1]. Amazon took this fact into account and is currently testing Prime Air, an autonomous drop-by delivery system [2]. Several companies such as Flirtey [3], DHL and Google also look at these vehicles as a mean of transport for parcel delivery services (e.g., package, food, medical devices). The evolution of deep learning led to the development of reliable real-time algorithms for object detection in imagery. Internet of Things (IoT) propelled the development of cheaper, smaller and more powerful hardware (i.e., processors and cameras). This led Computer Vision (CV) to be, presently, a reality in mobile devices and other portable products available in the common market as a useful or even required technology. These algorithms are trained and tweaked with datasets made of a gigantic volume of well-labeled images. This information is fed into deep-learning models to form an input evaluator that, if enough in terms of correctness and case exhaustiveness, results in an equally correct output. The better the dataset, the more trustworthy the results of the model [4].

**Figure 1:**
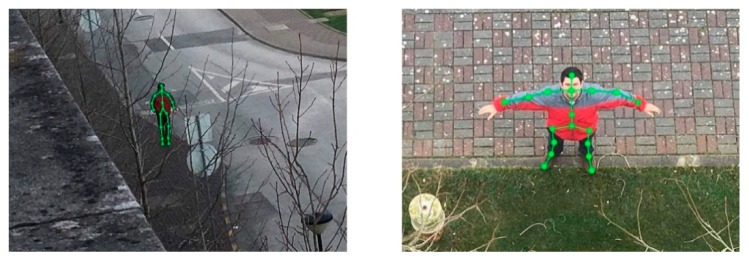
Example of skeleton mappings with different proportions.

Among the main problems of a camera dependent solution, the quality and clearness of the captured image occupy a privileged position thanks to motion blur, prop flicker, UAV vibrations, low spatial resolution itself and other environmental related limitations (e.g., solar light reflection, fog, humidity particles) [5,6]. The detection of a person from an aerial view becomes even trickier since different angles mess with the proportions and shape of the human skeleton (Figure 1). The out-of-the-box drones available in the consumer market and their hardware are usually inaccessible, which means that it is not possible, through the conventional ways, to embed a visual recognition system among the existent functionalities. Moreover, the components are not ready for the additional overhead of an extra task. Related to the image processing itself, visual recognition is a very onerous task, which means that to get the best performance from the deep-learning models we need a device with powerful computing power, an attribute directly proportional to the price of the equipment. The choice of the Deep Neural Networks (DNN) architecture and the respective model training are crucial to overpass this last challenge that is focused on performing human detection through a UAV equipped with a low-cost and low-performance computing device and camera.

Internet is almost a requirement in our mobile devices presently. Many software is being deployed to the cloud where it can be accessed, anytime and anywhere, which means that users have a wide variety of services available at the distance of a click. We took advantage from these facts and implemented a platform to deploy UAS-based real-time services, designed to be highly scalable considering different scenarios (e.g., racing, firefighting, surveillance) [7].

In autonomous delivery services drones may carry sensible and valuable goods. Therefore, it is important for the payload to be delivered near the person who is authorized for the reception, instead of landing or dropping the package in a predefined location. The definition of a static final drop point can be problematic. There can be changes in the disposition of the territory and the coordinates defined as landing zone can be the resting place of a new decorative prop that will be nothing more than an obstacle. The precision error of the location services (i.e., GPS) caused by signal interference can also result in the drone landing on an incorrect and harmful position (e.g., swimming pool, tree).

The existence of these many failure points originated by the total dependency on the GPS for the landing phase of a delivery drone creates the need to study how can this problem be overpassed. The solution of these functional holes can help bringing drones forward as part of our society. The high availability and performance of current CV techniques makes it possible to use the drone camera as a sensor for the estimation of the landing spot. It is a great opportunity to understand how the faulty GPS-based delivery can be supported by the processing of data acquired without extra equipment, since most drones already include a camera.

To improve the previously mentioned platform, in this work we propose a detection system that allows estimation of the location of a person, from an aerial perspective as part of a drone-based delivery service. The functioning of the system is supported by cost-effective low-performance equipment and uses CV and Machine Learning (ML) techniques. The objective is to estimate in real time the landing or delivery site around a predefined geolocation so that the drone can get closer and identify the potential receiver as the person who ordered the delivery. Besides solving the safety and authorization problems found in other delivery processes, this service is intended to be an out-of-the-box solution for people who live remotely and need urgent supplies such as meds or food. We can consider three phases in the delivery, beginning with the detection of a potential receiver, the respective approach to acquire the authorization through authentication and, finally, the landing. In this work, we focus on the detection of a person through images captured the drone’s camera to start the approach and identification of the receiver for authorization purposes. The sum up of the previous paragraph results in the following research question.

**Research Question:** “Is it possible to detect a person from an aerial perspective using a UAV and Computer Vision techniques in order to execute a safe landing with cost-effective and low-performance equipment?”

To answer the defined question, this work followed the next methodology. In the first step was the analysis of the current background about object detection and drone landing. It was considered the development areas and tools related to remote sensing (i.e., CV, ML, IoT) and the state-of-the-art work that connects it to UASs. Based on this research, an architecture was designed to fulfil the objectives of this work. The next step was to create a functional prototype to be iteratively evaluated along different test scenarios. For each scenario we collected and analyzed the results. The interpretation of the results of each test influenced the performance of the next assessment.

The development of this work leads to the following main contributions:

1. Identification and analysis of a problem related to UAS in a new generation service;
2. Study of the current technology available in the fields of CV and IoT that can be useful for remote sensing in this specific situation and proposal of a low-cost system to obliterate the current problems of autonomous delivery;
3. Creation of a new approach to detect a potential receiver during the landing phase of the goods delivery process;
4. Development of a functional prototype to test the proposed system;
5. Assessment of the solution, defining the best conditions for the proper person detection from an aerial perspective—the best heights, horizontal distances, day times, and algorithms’ confidence thresholds.

The last contribution allows understanding of how the performance of a low-performance device varies in human detection through CV. The obtained results are valuable to tune the parameters that influence the behavior of the system. The study of the declared hypothesis considering low-performance cost-effective equipment can help bring this type of task to low-end UAVs instead of limiting it to expensive equipment. The increased accessibility of an on-the-run human detection system can boost the development of delivery services and similar tasks, by reducing the cost of each fail-safe system.

This paper is the continuation of a previously published study [8]. In the previous work, it was defined the solution’s architecture and the prototype implementation. For the prototype’s assessment, it was performed one test scenario to validate the solution. This paper was originated, then, as an extension of the previous article. In this, we make a deeper research on the area, over the state-of-the-art research, and the prototype was improved. The first test scenario was repeated with the new prototype and there were executed four more test plans to complement the solution’s evaluation and assessment in different conditions and scenarios. The first new test scenario goals were to assess the performance of the solution on a real case, with a commercial drone. The next test case was made to compare the difference between a low-cost camera and a mid-range camera. Finally, the last two new tests had in mind the evaluation of the algorithms’ accuracy under different daytimes—morning, afternoon, and night—and the comprehension of the ratio between precision and recall values using different confidence thresholds. Performing these tests, we could find the best conditions (i.e., heights and horizontal distances) for a correct person detection to achieve the best balance between the precision and recall values.

The structure of this paper is as follows. In Section 2 we perform an overview of the background concepts related to this research area. In Section 3 we present a brief state of the art of visual recognition focused on object detection and DNN. Then, in Section 4 we list the requirements and analyze the architecture that supports the proposed solution. In the Section 5 the implementation of the prototype is described. Next, in Section 6 the assessment of the solution is presented and discussed for each of the five test scenarios created. Finally, the last section includes the conclusions and intended future work.

## 2. Background Overview

Visual recognition can be applied to digital imagery to obtain valuable information. Processing video in real time requires high-performance machinery to extract useful and reliable visual information in a feasible meantime. There are different needs regarding image analysis. For instance, image classification intends to answer what is depicted in the picture, while object detection answers where. Also, presently there are cloud-based solutions that allow the running of the onerous part of the image analysis process in powerful computers instead of local machines that can be low-performance devices (e.g., Google Cloud Vision, IBM Watson Visual Recognition, Microsoft Azure Computer Vision, Amazon Rekognition) [9]. In this case, a stable and fast Internet connection is required to send the media to cloud-based services to interpret it and send the output back to the client. Otherwise, the process needs to be performed locally by a device with more processing capabilities, working in Internet shadow areas as well. There are many fields benefiting from visual recognition. In retailing, Amazon Go corresponds to a store without employees with a technology called “Just Walk Out” that merges CV, deep learning and sensor fusion to identify and track the customer around the store and know which products were taken from the shelves [10]. In agriculture and botany there are systems that check if the food is proper for consumption (e.g., spoiled fruit detection) or detect diseases in plants [11,12]. Related to security and surveillance, there are CV systems to recognize license plates [13] or perform facial identification of individuals in crowds, through UAS-based platforms [14]. In autonomous driving and driving assistance, cars are now able to detect obstacles in the road and pavement marks and interpret the road signs (i.e., image classification) for speed maintenance, for instance [15]. Among many other areas, infrastructure control also benefits from deep learning allied to imagery to improve infrastructure resilience, such as railway inspection [4].

Besides the ready-to-use products that are available through the Internet, there are public resources for research and customization. In recent years, many algorithms and techniques have been developed to improve two of the most important performance measures: increase the accuracy and decrease the time/resources required to apply them. A priori, these techniques require the creation of a good dataset. In CV, datasets are fundamental for the recognition and identification of objects. Through a computerized process, data is extracted and converted into rules that, for an input, make an output prediction. Considering an image as input, the output can whether be a flag indicating if a specific object is present in the photo, what object categories are present (i.e., laptop, smartphone, person) or the specific location of each identified object.

The dataset creation starts with the gathering of pictures of different scenarios, containing the objects that are intended to be detected in the new inputs. Then, for each image it is necessary to create an annotation file with the available tools (e.g., Labelbox, Fast Image Data Annotation Tool, LabelImg, CV Annotation Tool). To achieve pleasant results on the prediction, it is advisable to collect a rich and large set of pictures, in different scenarios and with different variations. After the creation of the dataset, the pictures and the respective annotations are introduced in a DNN that will be trained based on predefined classification possibilities. It is common, for the evaluation of these neural networks, to split the pre-train dataset in two: one for training and the other to evaluate the model. Taking into account that the step of image capture and labeling is very time consuming, there are public datasets that can be used (e.g., SceneNet [16], ImageNet [17]). Moreover, the datasets with more people related information are INRIA Person [18] and Microsoft Common Objects in Context (COCO) [19] with 250,000 pictures of people.

**Figure 2:**
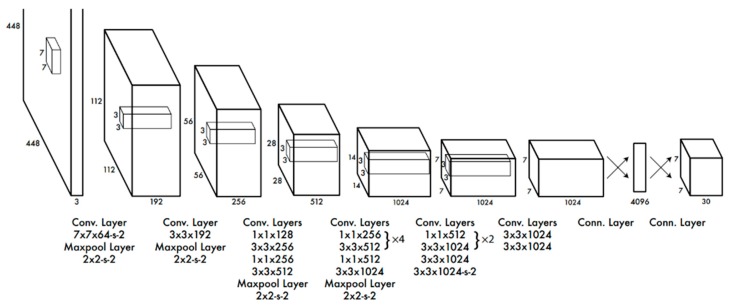
Architecture of YOLO, a single shot object detection model [21].

The first CV algorithms that appeared in this area consisted of analyzing the full images without almost no treatment or manipulation [20]. Applying an untreated input to the neural networks tends to be very exhaustive and time consuming [21]. Thus, a new method to perform object detection and recognition was created: Convolutional Neural Networks (CNNs). In CNNs, a specific set of operations is applied to the image input to prepare and extract its most important features. The preparation of the pictures differs for each model and is commonly based on convolution and pooling layers [22]. For instance, the input passes by one or more layers to create multiple outputs that highlight specific patterns (e.g., contours, color variations). The output of these layers is finally applied to the fully connected neural network layers to predict the score of each classification possibility. During the training and validation phases, the same procedures performed in the convolutional layers are applied before the processed of the data by the neural network. In the final, as the input is processed and normalized, the neural networks tend to be smaller, increasing the speed of the whole algorithm [22]. There are many different frameworks and models available to implement CNNs. The most significant differences between these algorithms are related to the number and type of the layers in the network’s architecture. The architecture of the You Only Look Once (YOLO) model is represented in Figure 2 [21]. This model is pre-trained with the ImageNet dataset to tune each of the layers according to the labels of the dataset. Among the known layers, the most common are convolutional and minimum, maximum, and average pooling. In the first, a convolutional matrix sliding is applied across the image, while the remaining reduce the size of the image by calculating the minimum, maximum or the average information of a group of pixels in each image area [4]. The difference in the number and size of each layer has a direct impact on the performance of the object detection process. Minding this fact, Google created the MobileNets framework, intended to run in embedded and mobile devices. This network can be used in different systems and presented better results when used with the algorithm Single Shot Detector (SSD) [23]. The combination of these two allows the creation of lightweight systems for object detection, deployable in embedded devices such as Raspberry Pi [24].

Briefly, the application of CV depends on two main factors: the acquisition of the imagery and its processing for interpretation. While the acquisition depends on the chosen camera, the last factor will vary in terms of the hardware and deep-learning algorithms used to process the interpretation of the media. Presently, near infrared, thermal, and depth-sensitive cameras can also be used for image recognition. However, the most common are RGB cameras, equipped with a sensor that collects the same bands of light as the human eye (i.e., red, green, and blue). Respecting the image acquisition and the processing algorithms, there are open-source tools that speed up the process. For instance, SimpleCV enables the developer to work with the received image or video stream, and the ML can be handled by TensorFlow, PyTorch, or OpenCV, a framework to perform both the tasks (i.e., image acquisition and data processing for interpretation).

## 3. Related Work

As previously mentioned, UAVs equipped with a GPS can use this technology for autonomous vehicle navigation. The precision of the GPS depends mainly on the number of satellite connections that can be affected by the presence of adjacent buildings or electromagnetic interferences [25,26]. Alternative solutions must exist to act when the navigation system fails during autonomous missions. The same happens relatively to the landing phase. To complement the GPS and solve these problems, new methods were developed using CV algorithms to predict and define the landing site.

Janousek and Marcon use a video camera and an IR-LOCK sensor to help in the landing step. In this work, a matrix of infrared (IR) emitters must be installed on the landing site [25]. The vehicle is sent to the GPS coordinates and, on a 10–15 m height, the vehicle’s IR sensor detects the emitters matrix and starts the landing phase.

To deal with indoor scenarios, where GPS signal is lost, Sani and Karimian proposed a low-cost navigation and landing solution, tested with the quadrotor Parrot AR.Drone 2.0 [27]. To achieve the required functionalities, they used the values from the Inertial Measurement Unit (IMU) sensor and the images of the camera. Using an ArUco marker, they define the landing area. This marker corresponds to 13 cm square with an internal binary matrix that represents a unique identifier. In the first step, the vehicle detects the marker with its camera and makes an initial approximation until it is almost aligned with it. They performed 10 tests and the average distance from the center of the UAV to the center of the marker was 6 cm.

Borowczyk et al. implemented a system that lands a UAV on the top of a car in movement [28]. On the top of the car it was placed an AprilTag marker [29] and a smartphone transmitting the GPS position of the car to the drone in real time. The UAV was equipped with a GPS receiver, a three-axis rotative camera and a wide camera pointing down that allows the visualization of the marker until the drone lands in safety. The performed tests were successful, and the drone could land in a car that was driving at a speed of 50 km/h.

Truong et al. [30] made a study of the existing solutions for the detection markers through CV. Based on the problems identified through their analysis, they also proposed a new method for detecting a marker for the landing phase of delivering goods using a UAV. The strategy was to enhance the low-quality images captured by a cost-effective ArduCam, with low spatial resolution, connected to a Raspberry Pi 3. The image is then directly fed into the module that combines super-resolution (SR) reconstruction and marker detection CNNs for detecting the landing area. This process is repeated until the UAV lands successfully on the ground. The obtained results demonstrated that the proposed solution had better performance than the other solutions analyzed.

In the article [31], Kim et al. proposed an algorithm that can land a UAV on a custom landing platform in movement. The images capture is made through an omnidirectional camera of a smartphone attached to the UAV. The landing site is marked with a red rectangle, and then they use a color filter algorithm to find the red contour in the camera frames. The system was validated using a quadcopter that landed successfully in a moving platform during outdoor tests.

Lee, Ryan and Kim proposed an algorithm based on Visual Servoing that uses a moving landing platform as a reference to the UAV [32]. To access the solution tests were performed in three different execution modes: Patrol Mode, IBVS-Guided Tracking Mode, and IBVS-Guided Landing Mode. In the Patrol Mode, the vehicle searches in the space until it finds the target platform and changes to the next mode: the IVBS-Guided Tracking mode. In this mode, the goal is to maintain the UAV stable, hovering 40 cm above the landing platform and then switch to the IBVS-Guided Landing Mode. This last mode is responsible for landing the vehicle on the platform if all the safety conditions are met. The developed work resulted in satisfying outcomes.

Nguyen et al. based their work on the poor experiences with the Amazon delivery service [33], proposing a new approach using markers to detect the landing site, without needing location systems. The onboard computer detects in real time the presence of a marker in different day times: morning, afternoon, evening, and night. They performed many test in different cases, proving that the solution performs better than the other ones in terms of robustness, precision, and processing time.

Zhao and Jiang presented a system that lands an AR Drone 2.0 autonomously [34]. In this case, they also used the AR markers for the recognition approach. After taking off, the detection system is automatically activated and starts finding the landing platform. For the detection process, the images are sent via Wi-Fi to a computer that handles the image analysis processing. Initially, the images are converted to white and black colors. Then, the algorithm finds the edges of the image to produce sets of four lines that can constitute a valid square marker. When it finally detects a valid marker, the vehicle lands on the top of that marker.

Wang, She, and Si developed another markers recognition algorithm that lands a UAV on a moving landing platform, composed by multiple AprilTag markers [35]. For the recognition it was used a monocular camera mounted on an anti-vibration gimbal. For the tests phase the authors used the drone DJI Matrice 100 equipped with a Zenmuse X3 gimbal and the Manifold onboard computer that permitted all the processing to be executed by the vehicle itself, without depending on offboard computers.

Barbedo et al. presented a case study that uses deep-learning models to detect animals in a farm, through aerial images captured from a UAV [36]. The goals for this work were to find the best distance for the detection of Canchim animals and what is the best CNN to get the highest accuracies for this problem. The footages were recorded with a DJI Phantom 4 Pro drone. The experience resulted in the analysis of 15 different CNN, and they conclude that all of them have enough robustness to detect the animals at 30 m altitude. For all the models, they obtained accuracies close to 100%.

Wubben et al. proposed a solution that can land a UAV, equipped with a low-cost camera, on an ArUco marker [37]. In the first phase, the UAV makes an initial approach to the landing site. Then, the detection system is triggered and starts looking for the markers. On the destination place, there were placed two markers with different sizes. The bigger marker should be detected from higher altitudes and then, when the vehicle goes down, it should detect the smaller marker to finish the landing procedure. For testing purposes, the authors used a hexacopter equipped with a Raspberry Pi 3 B+ and a Raspberry Pi camera for the images acquisition. Their solution could detect and land the UAV in the landing pad from a height of 30 m.

Nguyen et al. proposed a system to land a drone safely independently of the presence of GPS signal [38]. This is based on a marker, placed on the landing platform, and a visible-light-camera sensor. Throughout an image recognition algorithm, the vehicle detects the marker as the landing point. This algorithm, proposed by the authors, uses the LightDenseYolo CNN that extracts the information gathered by the camera and predicts the marker’s location. For testing purposes, they captured multiple videos with a DJI Phantom 4 quadcopter. Then, the neural network processing was ensured with external hardware, namely the software development kit Snapdragon Neural Processing Engine. Their solution demonstrated being robust in different environmental conditions, detecting the marker at different distances.

To sum up the analysis of the related works, we present a comparison in Table 1. They were compared by the usage of markers or not, the detected situation, the spaces where they can be applied and the detection method. The purpose of the first criterion is to understand if the solutions require the placement of markers to detect the landing place. The second criterion describes if the system can detect in movement or not. The third criterion identifies if the landing approach can be made only on indoor spaces, outdoor spaces, or in both. The following criteria describe the used method for detecting the landing site. Finally, the last three columns correspond to the descriptions, strengths, and weaknesses of each related work.

The goal of this work is to improve the process of goods delivering using drones, focusing on the detection of the potential receiver. The difficulties or limitations found in the compared alternatives should be obliterated complementing the GPS that is required to get to the destination point but can work with low accuracy thanks to different interference causes. We consider that one of the biggest failing points is the correspondence between the arrival location and the final landing spot. The idealized solution should consider the dynamic definition of the landing position in the moment of arrival, not exactly in the destination coordinates, but in the surroundings and depending on the receiver location. The use of a visual marker to define the landing position of the drone requires the presence of an additional element the user must place in a safe position. This practice becomes impracticable if we think that every new client subscribing a delivery service should receive or print its customized marker before the delivery of the parcel. This becomes even worse if every delivery company needs to use a different marker. Ideally, the receiver should need no additional elements to receive a package. On the other hand, it would be great if the drone would not require any additional equipment besides the common sensors to figure out its landing site, which leads us to the versatility of the camera. Through the analysis of the current technology and tools it is noticeable that there are many development opportunities in the area of CV, even for cost-effective low-performance devices. This means that it should possible for a UAS to detect a person (i.e., receiver) in the arrival location without expensive resources or additional equipment besides the processing device that can correspond to the flight controller itself, already present in the aircraft. This work can be done joining CV with deep learning to extract data from the images captured by a drone, regarding the receiver.

## 4. Architectural Proposal

CV plays an important role in turning this delivery system user friendly in a way that an individual should need no equipment (e.g., smartphone, Radio-Frequency Identification (RFID), visual badge) to be identified as the receiver and define the landing site. To perform the detection of the receiver through visual recognition, a camera is required. This camera and the processing equipment need to be low-cost and embeddable in a UAV. In terms of functionality, this system only needs to detect humans from an aerial view, in possible and diverse poses, in different types of environmental situations (e.g., snow, grass, dirt, road, bright, dark). The performance of the system should be balanced in a way that the drone does not take long to proceed its visual scan for humans if no one is identified in the iteration.

The delivery process of the idealized service depends on the presence of an individual, so there will be requirements regarding the user point-of-view. Among these requirements, the presence of the receiver on a safe place at the time of the delivery is necessary, as much as the attempt of getting in front of the drone to ease the detection process within a safe distance radius. Related to the same purpose, the person should not move around during the detection process.

**Figure 3:**
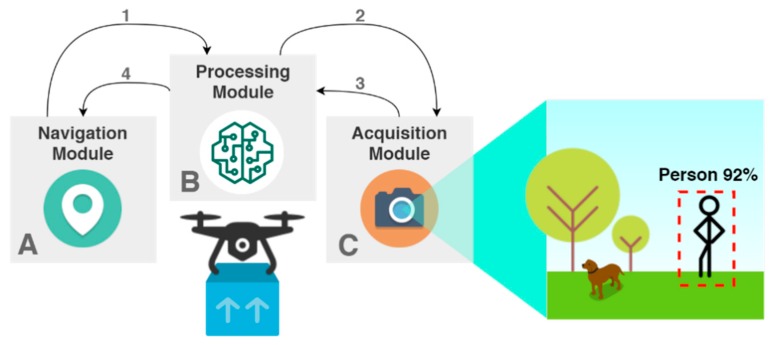
Architecture of the Human Detection System.

This system is made of three different modules, embedded in a UAV, that cooperate to make the human detection work as specified, through a camera and deep-learning algorithms (i.e., CNN), as represented in Figure 3. The first corresponds to the Navigation Module (A), responsible for autonomously flying the drone to a specified geolocation and perform punctual moves or rotations during the delivery phases. This autonomy is based on a mission planning that allow the setting of the course of a UAV to its destiny through GPS coordinates. Then, we need the Image Acquisition Module (C) that is made of a camera to capture images that are passed to the Processing Module (B), which is responsible for running the ML algorithms specified for person detection.

**Figure 4:**
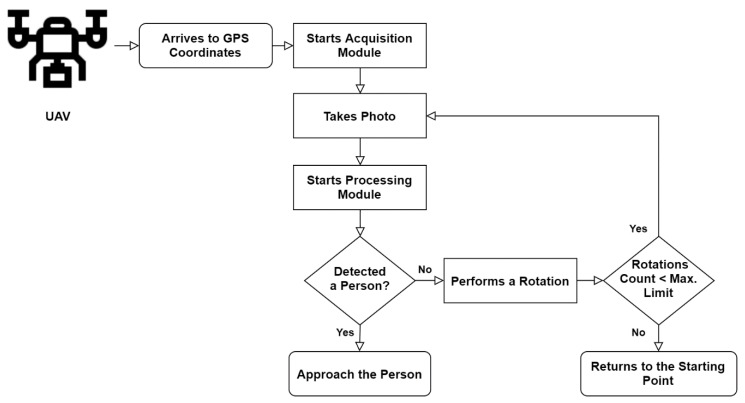
Architecture proposal block diagram, with the flow of the solution from the starting point to the approaching phase.

This architecture fulfils the objective of the proposed system, intended to work in the following scenario. When the UAV arrives to the GPS coordinates defined in the delivery mission as the receiver’s location, the Navigation Module notifies the Processing Module (1) to ask the Image Acquisition Module to turn on the camera (2). The first step corresponds to the acquisition of an image to be posteriorly handled by the Processing Module (3). Regarding the best-case scenario, the Navigation Module receives the location of a person to begin the approach and start the next phase of the delivery (4). On the other hand, if this module does not detect the presence of a person, the vehicle will rotate along its vertical axis until the Processing Module detects one person. The rotation interval depends on the Angle of View of the camera. This is performed cyclically until a potential receiver is detected. After performing a pre-determined amount of rotations, if the vehicle did not detect one person, it should go back to the home point and trigger an alert that it was impossible to find the delivery site. The flowchart of the solution is presented in the Figure 4, representing and describing the steps that the UAV accomplishes from the GPS coordinates definition until the person’s approach.

## 5. Prototype

This section describes the development of a prototype and includes the steps we took to get the human detection working on a quadcopter. Since most out-of-the-box drones available in the market are impossible to program and change through the conventional ways, the UAV to implement this system should be assembled modularly, as the one specified in a previous work [39], where the control of the drone is guaranteed by the autopilot NAVIO2 installed on a Raspberry Pi 3 microcomputer. The communication with a vehicle controller was specified in another previous work [40] that allows the system to interact and monitor a unmanned vehicle anytime, anywhere.

The prototype is composed by the microcomputer referred above, equipped with the Raspberry Pi NoIR Camera V2 with 8 MP resolution. This computer is a low-cost solution capable of running a Linux-based operating system, which fulfils the requirements for the installation of OpenCV 4.0 and TensorFlow, the essential technologies for the training and usage of the person detection algorithms.

**Figure 5:**
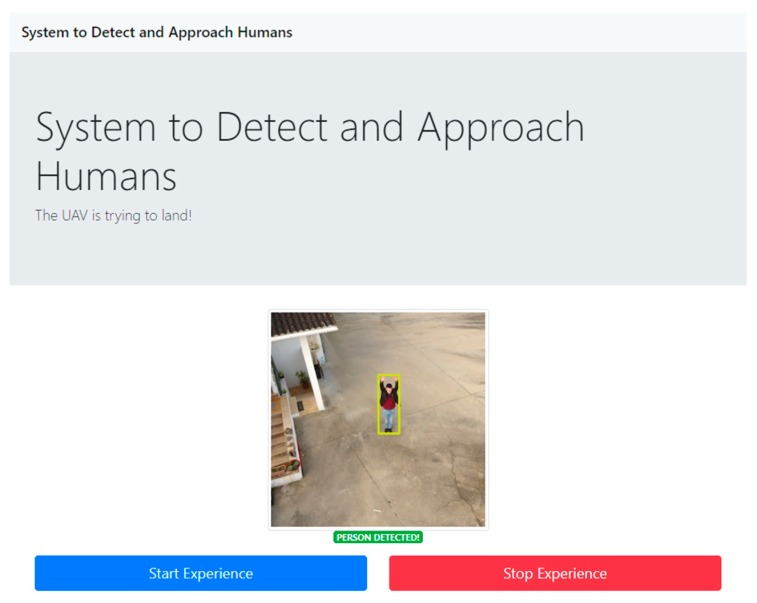
Screenshot of the application built to run the person detection prototype.

The purposed system consists of three different modules: navigation, acquisition, and processing. The development of the Navigation Module was not considered in this prototype, as we only focused on the delivery system. The responsibility of this module for this phase is to trigger the Processing Module when the vehicle arrives at the predefined GPS coordinates. At this step, the Processing Module starts the service for the images acquisition, handled by the Acquisition Module. During the implementation process, it was created a Python application to represent the acquisition and processing modules functionalities. To trigger the execution of the developed human detection system an application was built, represented by the application interface in Figure 5. This includes a representation of the captured images and two buttons to start and stop the process.

### 5.1. Implementation of the Modules

Relatively to the Acquisition Module, the access to the Raspberry Pi camera, to capture the images with a 300 × 300 px resolution, was made through a Python 3.5 script using the OpenCV 4.0 library. Then, we used the same library to manipulate and analyze the photos. The first step after the capture is to transform the color scheme of the image from RGB to a BGR (Blue, Green, and Red) matrix to fit the specifications of the OpenCV modus operandi.

The Processing Module is responsible for the load, execution, and processing of the person detection algorithm. In the first moment, we had to choose the most appropriate CNN model according to our requirements (capable of running on a small-powered ARM architecture device). Based on the research made in the section that analyzes the state of the art, we chose the algorithm SSDLite, a lighter version of SSD, in conjunction with the feature-extractor MobileNet that Google created with the aim for Android mobile phones based on ARM architecture. According to a survey paper to study the tradeoff between speed and accuracy for Faster R-CNN, R-FCN, and SSD, concluded that SSD with MobileNet has the highest mean Average Precision (mAP) among the models targeted for real-time processing [41].

Since the training step of the model requires either time and a rich variation and a huge volume of pictures, we started with a pre-trained model on the COCO dataset that has 250,000 people identified [19]. This pre-trained model is available to be used directly on the TensorFlow framework. Although as we are already using OpenCV for the image acquisition step, we opted to convert the model to an OpenCV compatible format to use in this framework avoiding the need to load two different libraries in the script. After each iteration of an image processing, the model outputs the prediction for each classification label with its confidence and the area location of each object. For this prototype, we are only considering the label “person” with a higher confidence than 0.5 (50%), because there is more probability that a person really exists over detecting another object as person. This value is a starting reference and may be tuned, after the application on a test scenario, to avoid the detection of non-person elements.

### 5.2. Algorithm to Estimate the Person Location

Our main goal for this system is to approach the person waiting for delivery by using CV techniques. Accordingly, we will use the output of the Processing Module to estimate the presence and position of the person and go in his direction. Since quadcopters can rotate around their vertical axis, we will predict the angle of rotation to center the person with the front of the vehicle according to the following formula (1).

(1) $$\theta_{rotation}=\;-\;\frac{\left(\frac{x_{cameraResolution}}{2}\;-\;x_{centerOfPerson}\right)\;\times \;\theta_{horizontalAngleOfVision}\;}{x_{cameraResolution}}$$

**Figure 6:**
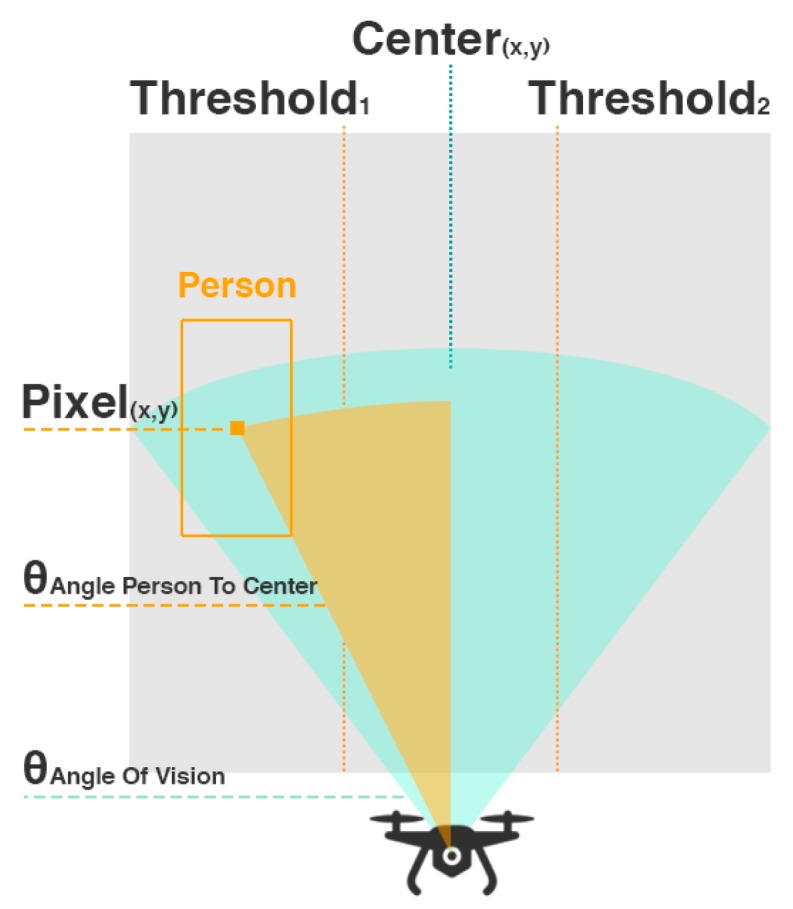
Representation of the horizontal angle vision of the camera.

This equation represents the estimation of the angle of the person ($\theta_{rotation}$) relatively to the horizontal center of the camera in degrees from the following input: camera resolution in the x axis (in pixels), the x value of the center of the person (in pixels), $x_{centerOfPerson}$ and the horizontal angle of vision of the camera (in degrees). As we know that $x_{cameraResolution}$ pixels correspond to $\theta_{horizontalAngleOfVision}$ degrees and we are close enough to the person based on the predefined GPS coordinates, we can infer the angle between the center of the person and the center of the UAV (Figure 6).

## 6. Test Scenario and Results

To evaluate the feasibility and performance of the detection process under different conditions we created an assess plan divided in five test scenarios. Therefore, in this section, we present the main goals of the testing phase and explain how the evaluation of the system was planned, executed, and analyzed, summarized in Table 2.

**Figure 7:**
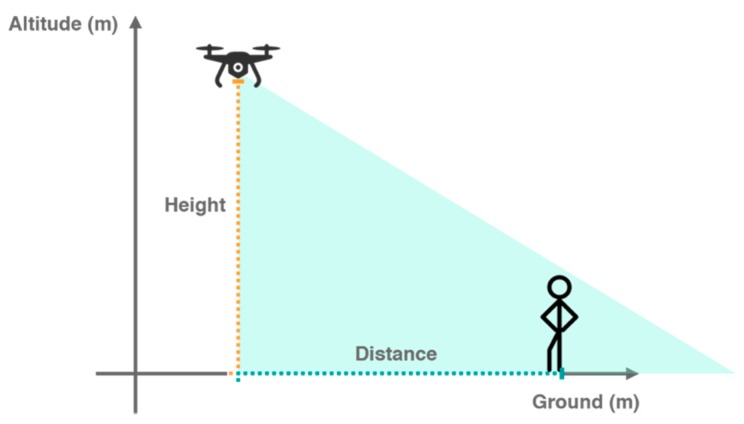
Explanation of the height and distance values described in the tests.

The first iteration of tests should primarily evaluate the correct behavior of the algorithms in terms of analyzing the pictures and detecting humans in them. Based on the analysis of different CNN models we opted to use the algorithms that are proper for the devices with ARM architecture, the SSD, and the SSDLite, which is meant to be a lighter version of the first one. The models’ full description and hyperparameters are detailed in the article [42]. These tests also contribute for the evaluation of both models to confirm which one offers the best results, running on a Raspberry Pi 3, for the precision, recall, confidence, and timing parameters. In each test scenario we considered the camera in different heights and horizontal distances as presented in Figure 7.

After getting successful results in these tests, the onboard factor was introduced through the capture of aerial footages with a UAS. From this perspective, it is possible to do more exhaustive tests on specific heights to understand the limits of the solution for the distances between the vehicle and the human. In these tests we used a DJI Phantom 4 that is equipped with a mid-range camera supported by an anti-vibration gimbal system pointed downwards with a 45 degrees positive inclination around the transverse axis. The processing of the image for the analysis of the respective data was done with the same prototype developed with the microcomputer Raspberry Pi 3.

Finally, as the final objective was to implement a solution entirely based on the Raspberry Pi 3, it and the respective Pi NoIR Camera V2 were fixed to the same DJI Phantom 4 with the same orientation configuration (i.e., 45 degrees facing downwards). This approach allowed us to present a final solution using a custom low-cost processing device and camera. For this test, we wanted to find the limits of the customized system, according to the distance and height values. Based on the obtained results, we could discuss the best balance between the accuracy of the model and each distance, to compare it with the previous test scenario that considers a high-quality camera.

For all the test scenarios we registered and evaluated the same metrics, through images resized to a resolution of 300 × 300 px, according to the expected input size of the processing algorithms. The first important value to interpret is the number of True Positives (TP). These values represent the people that were detected correctly. The second metric is the number of people that were not detected, known as False Negatives (FN). Then, the False Positives (FP) present the number of elements that the algorithm defined as humans incorrectly. For the TP values, we calculated the average confidence of the algorithm detecting a person correctly. Following, the precision and recall metrics are calculated as unit intervals. Precision indicates if the identified objects are truly persons while recall measures if the algorithm found all the persons in the pictures. The precision of the algorithm is calculated through the division of the number of persons identified correctly (TP) by the total objects identified in the photos (TP + FP) and the recall is calculated through the division of the number of persons identified correctly (TP) by the total humans in the photos (TP + FN). Finally, our goal also was to evaluate the performance times (i.e., duration of the processing stage), so we calculated the average elapsed time. The formulas for the total number of people (2), average confidence (3), precision (4), recall (5) and average time (6) are presented below.

(2) Nº of People=TP+FN

(3) $$Average\;Confidence=\frac{SUM\left(Confidence\;for\;All\;TP\right)}{TP}$$

(4) $$Precision=\frac{TP}{TP+FP}$$

(5) $$Recall=\frac{TP}{TP+FN}$$

(6) $$Average\;Time=\frac{SUM\left(Time\;for\;All\right)}{Samples}$$

**Figure 8:**
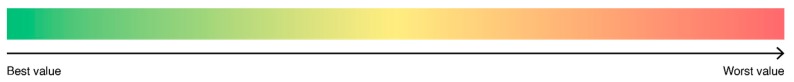
Gradient that represents the colors used as visual interpretation for the TP and average confidence values represented through tables. From the left to the right, the quality of the values decreases—the leftmost color (i.e., green) represents the best results, while the rightmost one (i.e., red) corresponds to the worst value.

In the analysis of the tests, some of the results are represented through tables. In the tables that show the TP and average confidence values, the color map in Figure 8 is used as visual interpretation of the data presented in each cell.

### 6.1. First Scenario: Tests and Results

The first scenario comprehends the tests for the validation of the implementation in terms of functionalities and was exposed to an exhaustive environment with many different elements, including the presence of cars, traffic signals, trash bins, and multiple persons. In this scenario, we acquired the images with an iPhone 6S Plus and used a Raspberry Pi 3 for posterior processing. The pictures were captured from the first and second floors of a building with heights of 4.5 and 10 m, respectively. In this situation, it was possible to attest the purpose of the algorithms and perceive the variation of the two models, SSD and SSDLite, according to the different heights, distances, and environments.

The Table 3 and Table 4 present the results obtained for the 4.5 m and 10 m heights, respectively. The first five columns of the tables indicate the height of the camera (in the y axis), the approximate horizontal distance between the individuals and the camera (in the x axis), the used model, the number of obtained samples, and the number of people present in all the pictures. Then, there are the values of TP, FP, FN, average confidence (Avg. Conf.), precision, recall and average elapsed time (Avg. Time) for each height and distance combination. The True Negatives (TN) were not considered because we could define that any non-person element was not identified as person.

### 6.2. First Scenario: Discussion

The Table 3 and Table 4 contains the results of the tests for different horizontal distances between the drone and the target people (i.e., 5, 10, 15, and 30 m) and at two different heights (i.e., 4.5 and 10 m) By analyzing the average time of processing between all the different scenarios, we can conclude that, independently of the height and distance, across each model we got similar processing intervals. The lighter version of the model could finish each step in less than half of the time of the original SSD version (~1.97 vs. ~4.66 seconds).

**Figure 9:**
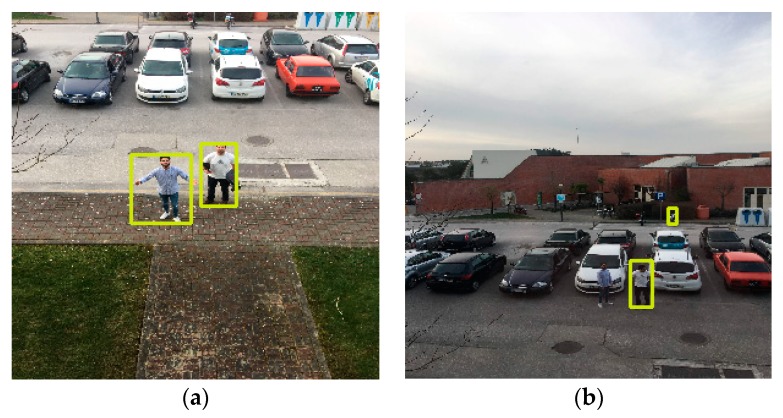
Person detection result at 4.5 m height: (**a**) 5 m distance; (**b**) 10 m distance.

When the drone’s camera was placed at a height of 4.5 m, the 5 m distance resulted in recalls of ~71% and ~94% for the SSD and SSDLite models, respectively, and precisions of 100% and 80% (Figure 9a). Increasing the distance to 10 m, both models highly decreased the value for recall (Figure 9b). The SSD model, in this case, got a recall of ~14% while the SSDLite detected people with ~29% recall. Meanwhile, the precision of SSD kept on 100%, meaning no false positive, while SSDLite reduced to 78%. For the longer distances, 15 and 30 m, SSD dropped the recall values to below 3% while the SSDLite achieved around 11%. The precision of SSD was always greater or equal 50%, while SSDLite shown precisions down to 39%. These values for both models can be explained by the dynamic elements in the environment, such as cars, traffic signals, trash bins, and people beyond the two targets.

**Figure 10:**
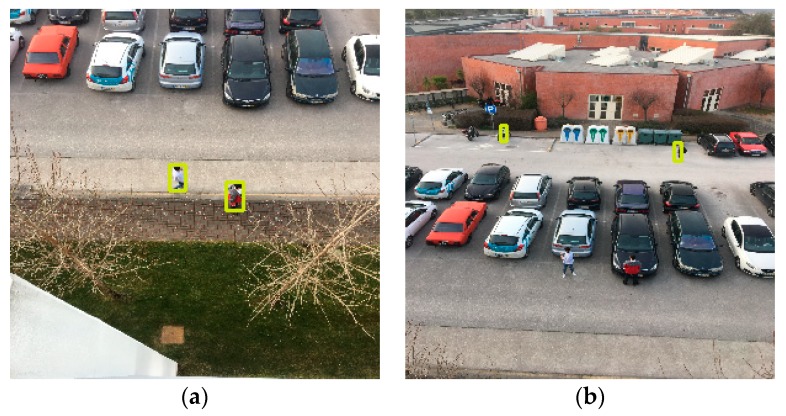
Person detection result at 10 m height: (**a**) 5 m distance; (**b**) 10 m distance.

For the second floor, 10 m height, we noticed a slight performance decrease in both models. The SSD recall dropped around 12% for the 5 m distance and the SSDLite recall dropped ~17% for the same conditions (Figure 10a). For the further distances, both models performed weakly, obtaining recalls below 8% (Figure 10b). In these cases, SSD presents better recall values than SSDLite by around 2% for each case. For the 30 m the values are slightly better, comprehended by the cleaner scenario where the target people were not between the cars. The precision values were, once more, far better on the SSD than on SSDLite: the differences between both models vary between 25%–58% for the same heights and distances.

Generally, the SSD presents lower recall and higher precision values than the SSDLite. While SSDLite identified 133 FP, SSD only identified 6. Associated with these values, we can also see that the average confidence of the SSD is higher in most cases. We can conclude that despite the SSD has lower recall, when it detects one positive value it is more precise and confident about it than the SSDLite, which detects more positives but with lower confidences.

### 6.3. Second Scenario: Tests and Results

**Figure 11:**
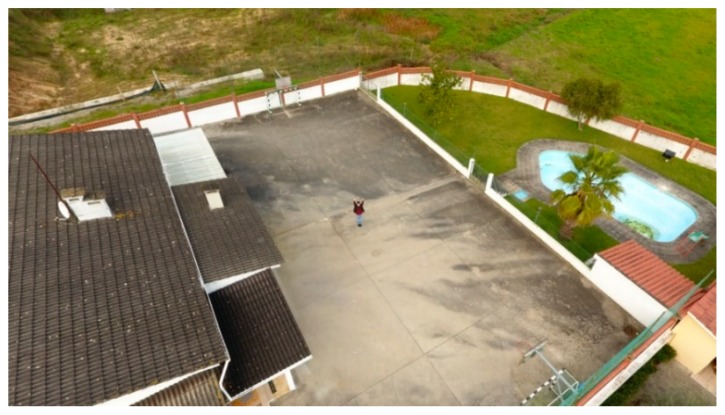
Yard of the house in the second and third test scenarios.

**Figure 12:**
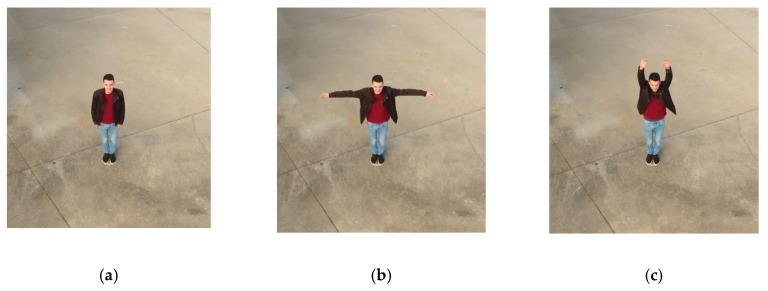
The three different poses for the second and third test scenarios: (**a**) arms down; (**b**) arms open; (**c**) yelling.

Following the first tests, we developed an onboard solution. The primary goal is to replicate a real scenario with a flying drone capturing photos from an aerial perspective. For this purpose, it was used a DJI Phantom 4 quadcopter with a built-in camera fixed on a stabilizer gimbal. Then, all the pictures were processed by the Raspberry Pi microcomputer. To represent a real environment, the pictures were captured on the yard of a house with one person (Figure 11). For each height and distance, we captured three samples corresponding to different poses, which are represented in Figure 12. The poses correspond to the person with the arms down (a), the arms open (b) and with the yelling to the vehicle (c). All the samples, captured with a 16:9 proportion, were cropped to represent a 1:1 aspect ratio and resized to 300 × 300 pixels.

**Figure 13:**
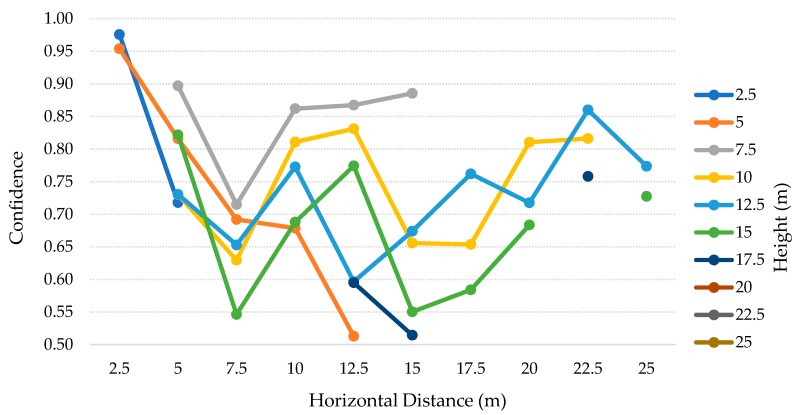
This graph represents the average confidence for the SSD-MN-V2, according to the horizontal distance between the UAV and the target person and the height from the ground. The values concern the second test scenario.

**Figure 14:**
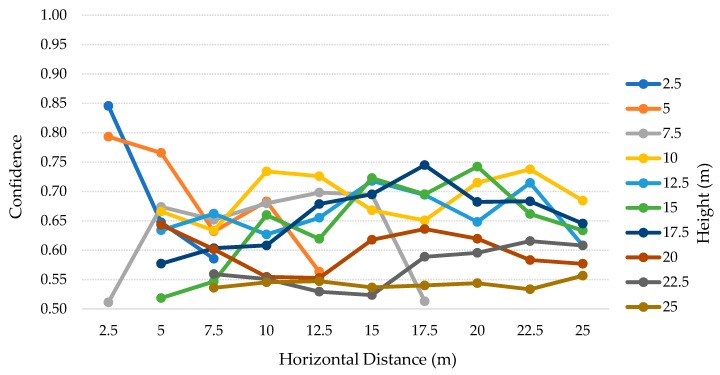
This graph represents the average confidence for the SSDLite-MN-V2, according to the horizontal distance between the UAV and the target person and the height from the ground. The values concern the second test scenario.

The results for this test scenario are presented in the Table 5. For this scenario, we considered both the height and distance up to 25 m, with steps of 2.5 m (2.5–25 m). Then, for each height we visually checked each of the images to see if the person was present. This means that we do not consider the person if the body is out of the camera range (represented as OR) or if the person is just partially present. The last case is represented as Incomplete Person (IP). Therefore, in the best-case scenario, minding the number of different heights, we can count with 30 positives (i.e., three poses for each of the 10 distances). Moreover, in some specific IP cases, the algorithm detected the person as a TP, and so we considered those situations as normal, instead of IP. The total positive values present for each height is represented in the total column, followed by the number of correctly identified people (i.e., TP) and the difference between the two values (i.e., FN). In the end, the precision and recall for the respective height are calculated. The TP and average confidence for each distances are detailed in the Table 6 and Table 7 and Figure 13, respectively, for the SSD model. On the other hand, the Table 8 and Table 9 and Figure 14 represent the SSDLite model.

### 6.4. Second Scenario: Discussion

The Table 5 contains the tests’ results for each height, from 2.5–25 m with 2.5 m steps, for both SSD and SSDLite models. Analyzing these results, we can observe that SSD, at a 2.5 m height, detected the person in the two distances where it was detectable (six true positives of a total of six individuals in the footages). In the Table 6, we can see that for the 2.5 m height the person was just present in the cases where the horizontal distance was 2.5 and 5 m. At the heights from 5–12.5 m, the recall dropped from ~9% up to ~30%. At the heights of 15 and 17.5 m, the recall is below 50%, resulting in ~41% and ~19% respectively. In the remaining heights, SSD model did not detect any person. Apart from these heights, this model achieved 100% precision in all the cases.

The SSDLite model got 100% recall for the heights of 5, 10, and 17.5 m. Still, the other heights up to 22.5 m presented recalls above 85%, decreasing on the 25 m to about ~71%. Though, from 15 m height the algorithm introduced multiple FP values and thus dropped the precision that settled between 50%–58%. After visually checking the photos, we saw that a basketball table in the yard was often detected as a person.

Interpreting the Table 6 and Table 7 and Figure 13, for the SSD model, we can find interesting patterns and correlations between heights and distances. For the lower heights, the vision field is reduced, and the camera can just observe up to 5 m distance. When the person was furtherer, it became not present in the photos. As the height increased, the vision field became wider and the number of OR and IP cases decreased to one, from 12.5–20 m. However, above 10 m the number of TP is inferior, with high impact above 15 m. Along with that, we can also perceive a strong relation between the average confidence and the number of TP. In most cases, on the higher values for TP, the average confidence is also higher. Moreover, there is a tendency to get 100% recall (three TP, for the three poses), when the height matches the horizontal distance or is very close to matching. This pattern can be explained by the positioning angle of the camera (i.e., 45 degrees).

The SSDLite results are presented in the Table 8 and Table 9 and Figure 14. The OR cases are the same, while the IP cases were detected even with low confidence values. This model detected at least one pose for all the height-distance combinations where a person was present. Moreover, the overall confidence values for this model are lower than the SSD ones. Once more, as stated on the previous test scenario, SSD achieved higher precision values, meaning that no FP have been detected, while SSDLite found more TP but introduced the detection of non-person objects.

### 6.5. Third Scenario: Tests and Results

**Figure 15:**
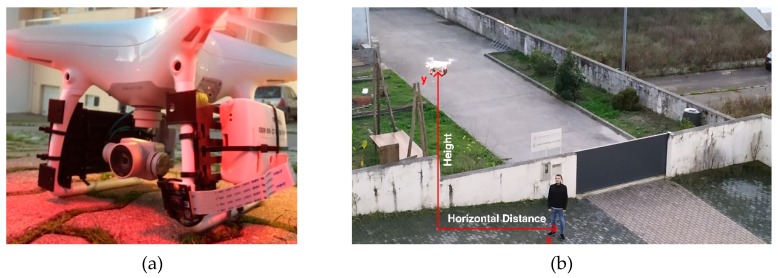
(**a**) Picture of the DJI Phantom 4 with the Raspberry Pi 3 microcomputer, camera and the power bank; (**b**) representation of the third test scenario, with the drone carrying Raspberry Pi.

The next tests were performed in the same yard with different camera equipment. Since one of the objectives of this work is to implement a low-cost solution, we replaced the DJI Phantom 4 camera with a Raspberry Pi NoIR Camera V2. The tested prototype (i.e., Raspberry Pi 3) was attached in the same DJI quadcopter, connected to the Pi Camera and a power bank to provide enough power for the coupled system, as seen in Figure 15.

This way, we can compare both mid/high-end range to this low-cost alternative and understand the benefits and cons of each scenario. The samples were captured directly with the size of 300 × 300 pixels by the onboard Raspberry Pi, triggered via the Python script implemented and explained in the section that describes the prototype.

**Figure 16:**
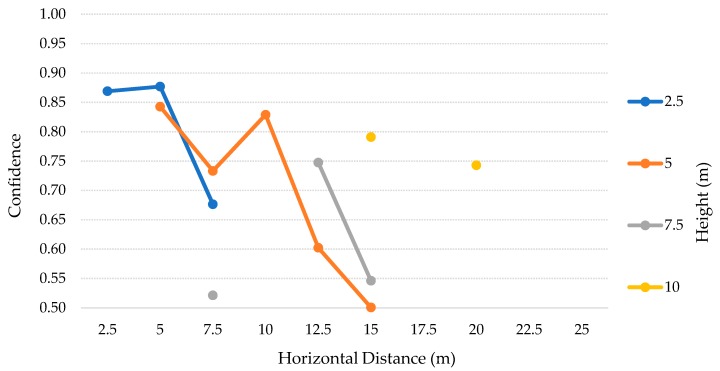
This graph represents the average confidence for the SSD-MN-V2, according to the horizontal distance between the UAV and the target person and the height from the ground. The values concern the third test scenario.

**Figure 17:**
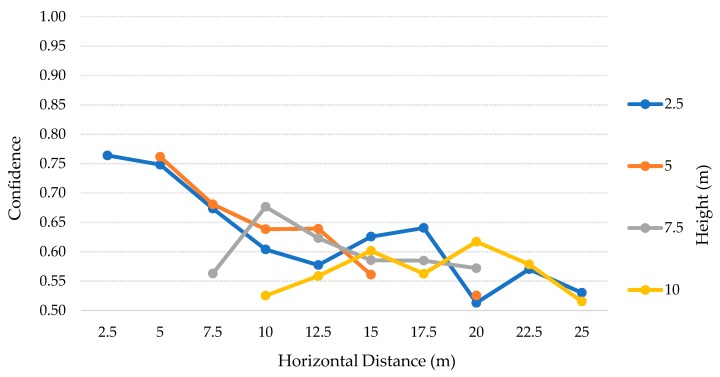
This graph represents the average confidence for the SSDLite-MN-V2, according to the horizontal distance between the UAV and the target person and the height from the ground. The values concern the third test scenario.

The results for this test scenario are presented in the Table 10. For this scenario, we decided to stop at the height of 10 m and horizontally move away up to 25 m, with steps of 2.5 m, considering 2.5–10 m and 2.5–25 m, respectively. The 10 m height limit was chosen according to the discussion of the previous test scenarios, where we concluded that this height was the limit for the best results, regarding the precision, recall, and FP values. The procedure for calculating the values was the same used in the second test scenario. Therefore, the TP values and the average confidence for each height and distance are detailed in the Table 11 and Table 12 and Figure 16, for the SSD model. On the other hand, the Table 13 and Table 14 and Figure 17 represent the SSDLite model.

### 6.6. Third Scenario: Discussion

The Table 10 contains the results of the tests for each height, from 2.5–10 m with 2.5 m steps, for both SSD and SSDLite models. Analyzing these results, we can notice that SSDLite has better recall results than SSD for all the heights. The highest recall for SSD is ~33% at a 5 m height, while the SSDLite peak value is ~76% at 10 m height. However, this was the unique case in which two FP were detected, decreasing the precision from 100% to ~89%. The second-best result for SSDLite was at a 2.5 m height, with ~70% recall.

According to Table 11 and Table 12 and Figure 16, this low-cost camera can comprise more distances at lower heights and stops reaching the closer distances right from 5 m height. Nevertheless, for 10 m height and above it detected only up to one TP in seven cases out of 25, all of them with considerable low confidence values. The best results for this model were in the lower heights and distances, where it could detect almost all the TP with great confidence, above 74%.

The SSDLite results are presented in the Table 13 and Table 14 and Figure 17. Apart from the same OR situations, this model could detect the person on the 10 m height and distance IP case. The SSDLite model detected 70 TP, while the SSD only detected 22. The problem we discussed before related to the SSD from 10 m height up is partially solved by this model, which detected at least one person in 23 cases out of 28. As expected thanks to the conclusions of the previous tests, even though the SSDLite finds more TP, the confidence of the predictions is lower.

Comparing the results of the second test scenario with these ones, we can deduce that for the same heights, the Raspberry Pi camera can capture the person in more distances than the DJI’s camera. On the other side, when the drone is at higher altitudes, this solution does not have enough visual range to analyze the closer cases. For example, at a 10 m height the low-cost solution has three cases of OR while the DJI solution has just 1. Despite the wider vision angle at lower heights with the Raspberry Pi camera, SSDLite model could get more TP in these cases, even though the confidence values were close to 50%.

### 6.7. Fourth Scenario: Tests and Results

**Figure 18:**
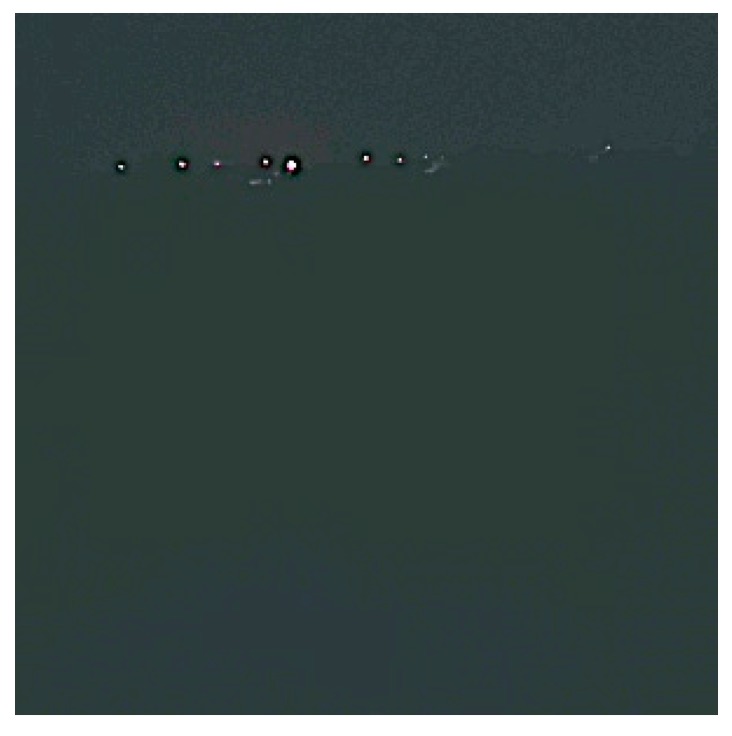
Image captured in the night, with the Raspberry Pi NoIR camera. The person is not visible at all, and only bright lights and contours are noticeable.

In this scenario, we intended to analyze and comprehend how the different daytimes can affect the accuracy of the solution. Despite this solution is focused on morning and afternoon times, the tests included, on the first stage, a night scenario. However, due to the equipment used and explained above, these results were not quite good, mainly black, and were not considered for these tests’ evaluation and discussion. The Figure 18 represents one of the night captures. Even with saturation and brightness changes, only the streetlights could be detected and evidence of contours on the landscape.

For the following analysis we replicated the third test scenario, with the Raspberry Pi camera, for the same heights (2.5–10 m) but only up to 15 m distances. This assumption was according to the third test results, where both models performed well up to that distance, and suffered high negative impacts above—for instance, SSD only detected one more person in all the distances from 17.5–25 m and, with SSDLite, this distance was the last with 100% recall.

The summary results are organized in the Table 15, with similar metrics used in the second and third tests, without the average accuracy and with the daytime. The morning tests more precise values are detailed in the Table 16 and Table 17 and Figure 17, for the SSD-MN-V2 model, and in the Table 18 and Table 19 and for the SSDLite-MN-V2. Finally, the Table 20 and Table 21 and present the values for the afternoon tests, using SSD-MN-V2 model, while Table 22 and Table 23 and present the values for the SSDLite-MN-V2 model.

### 6.8. Fourth Scenario: Discussion

In both daytime phases, the SSD-MN-V2 model predicted the presence of a person with 100% precision. In other way, SSDLite-MN-V2 had lower values for the 5, 7.5 and 10 m heights during morning and 10 m height during afternoon. The recall was substantially better on the afternoon conditions—SSD performed recall values from 0%–44% on morning versus 16%–60% on afternoon, while SSDLite performed values from 16%–60%, during morning, and 77%–100% during afternoon.

Considering the results obtained, a pattern is noticeable between the two different daytimes. The morning daytime did present lower accuracy than the afternoon condition. In the morning, the SSD did not find persons for the 7.5 and 10 m height, while in the afternoon it just did not detect on the 10 m height. SSDLite, though, found TP values on all the heights, despite having lower accuracy in the morning. One of the justifications we concluded with these results comprehend the different brightness conditions that can obfuscate the person or reduce the contrast between the person and the environment (e.g., in the morning, the sun light is brighter than in the afternoon).

### 6.9. Fifth Scenario: Tests and Results

For the tests we did before, there were only considered the positive values with confidence equal or higher than 50%. This assumption was considered on a negative-positive balance—with 50% or higher confidence, it means that the algorithm is more sure than unsure that it is a True Positive prediction. However, in this last test scenario, we took in account different thresholds for the confidence to understand the precision and recall values evolution when the minimum confidence is reduced to 10% and maximized up to 90%, with 10% steps. Associated with the respective precision and recall values, there are formulas intended to assess the best ratio between them. To measure and find the best ratio it is commonly used the Fβ-Measure, also known as Fβ-Score (7). This calculation is based on the precision and recall values and a beta (β) multiplier that preponderates the weight of precision over recall—the F1-Measure is calculated when FP and FN are equally cost; the F2-Measure is calculated when FN are more costly than FP; F0.5-Measure when the FP are more costly than FN [43]. In our solution, it may be critical to detect a non-person object as a person (FP), and so we calculated and present the F0.5-Score.

(7) $$F\beta \;Score=\left(\left(1+\beta^{2}\right)\times Precision\times Recall\right)/\left(\beta^{2}\times Precision+Recall\right)$$

**Figure 19:**
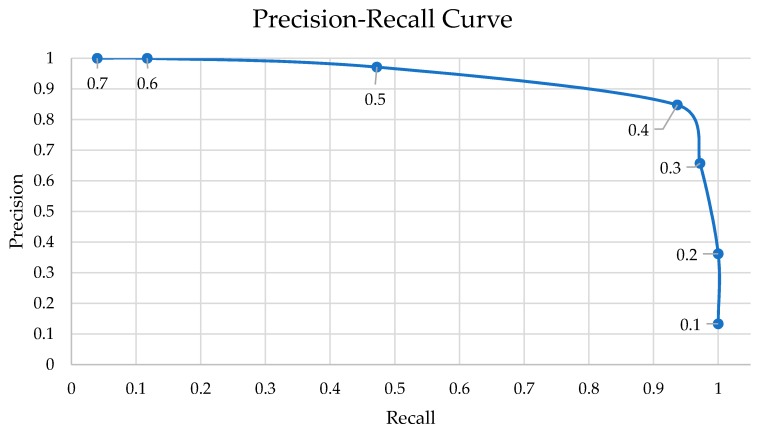
Precision–Recall curve for the fifth test scenario, indicating the ratio between the precision and recall values for different threshold values: from 0.7 to 0.1—left to right.

This test scenario is based on the third test cases, by using the Raspberry Pi NoIR camera V2 and the SSDLite model, for the same heights and distances—i.e., 2.5–10 m heights and 2.5–25 m distances. The summarized results are detailed in the Table 24 and in the Figure 19 we present a Precision–Recall curve, which is helpful to comprehend the decrease of precision while the recall values get higher. The evolution of the curve means the increasing of the threshold value, resulting in lower precision but higher recall values. In other words, the number of detected persons increases, while the number of non-person objects also increases.

### 6.10. Fifth Scenario: Discussion

**Figure 20:**
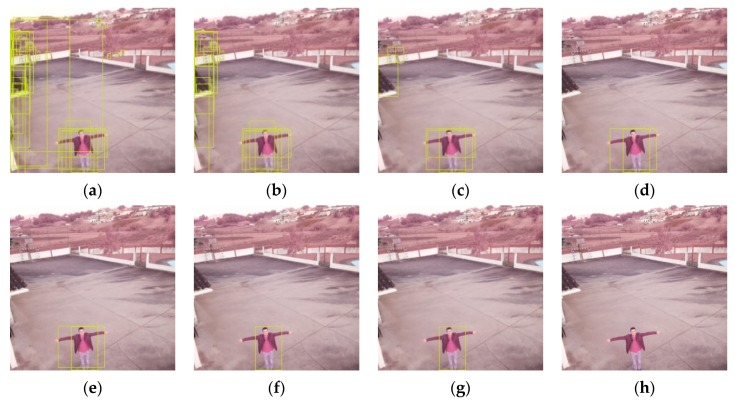
Prediction results for different confidence thresholds, identified by yellow rectangles: (**a**) 0.10 threshold; (**b**) 0.20 threshold; (**c**) 0.30 threshold; (**d**) 0.40 threshold (**e**) 0.50 threshold; (**f**) 0.60 threshold; (**g**) 0.70 threshold; (**h**) 0.80/0.90 threshold.

There are significant differences on these tests results (Figure 20). For a 0.80 threshold and higher (Figure 20h), the algorithm did not find any person in the pictures. For the 0.60 (Figure 20f) and 0.70 (Figure 20g) the precision was 100%, which means that there were not detected any objects that were not persons. However, the recall of these cases is significantly low—there were detected at most 36% of the total of persons in the photos. The recall value almost doubled to the 0.50 confidence (Figure 20e), while two FP appeared. For the 0.40 confidence threshold (Figure 20d), there appeared more four FP and the recall increased 0.19. The lower thresholds (i.e., 0.10–0.30—Figure 20a–c) presented high number of FP, and so should not be considered in any case for this solution. This fact explains the higher recall values, but strongly low precisions.

Facing the results, the better threshold values reside near the 0.50 confidence mark. This is a turning point where there are introduced just a few FP while keeping a recall of 0.66, meaning that it detected more than a half of the persons in the pictures. Analyzing the pictures one by one, we detected that the two FP values were in the same photo, in which the person was not present.

## 7. Conclusions and Future Work

Autonomous delivery is a topic that can bring UASs to our daily activities in a very close future. This kind of task depends on the GPS signal to drop the package in the predefined coordinates. However, there is a chance that an approach totally dependent on the location technology can be faulty. Besides the possible GPS errors related to signal interference or low precision, the surroundings of the fixed delivery point can be inadequate for the drone to land.

In this work we propose a solution that performs human detection from an aerial perspective through techniques of CV with the objective of estimating a safe landing location near the potential receiver. The integration of this solution in a UAS allows the filling of the lacunas found in the landing phase of the delivery process. Also, the focus of this research is on low-cost equipment (i.e., camera, processing device) to understand if the solution can be cost-effective. The proposed system adds to the GPS the techniques of CV based on DNN to detect a human standing in the delivery area. This considers a situation where the drone arrives autonomously in the delivery location through the GPS, according to the target coordinates defined in the system. When the vehicle arrives in the destination area, the solution executes its detection system to find a potential receiver. On its turn, the receiver should be facing the drone and marking the supposed safe landing spot just by standing in position.

The designed architecture is divided in three modules: navigation, acquisition and processing. While the navigation deals with the flight of the UAV, the acquisition is responsible for acquiring the imagery. On its turn, the processing runs the detection algorithms over the images to find the person. It also manipulates the captured picture and submits it across a DNN with a MobileNet V2 SSDLite architecture, pre-trained with the COCO dataset.

The solution was tested iteratively in five different contexts that converge in the final prototype. This prototype consists of a device with low computing power (i.e., Raspberry Pi 3) and a cost-effective camera (i.e., Pi NoIR Camera V2) that are fixed to a commercial drone (i.e., DJI Phantom 4). The first test was made to analyze the performance of Raspberry Pi 3 in the processing of the detection algorithms. The dataset is based on pictures captured with a smartphone from different levels of a building and the CNN models tested are the SSD-MobileNet-V2 and the SSDLite-MobileNet-V2, designed for devices with low computing capabilities. In the second scenario the pictures are captured through the camera of a commercial drone, positioned with a predefined positive rotation of 45 degrees around its transverse axis. This acquisition approach represents the real case of a UAV perspective and allows understanding of the more adequate distances, between the aircraft and the potential receiver. The difference between the first two and the last scenarios is that in the latter acquires the pictures with cost-effective equipment. This test allows us to understand how the quality of a low budget camera affects the performance of the developed human detection system.

For the first test scenario, the pictures are captured from two different heights in a complex environment with people and other elements of different types. While the SSD model took around 4.5 s to process each image, the SSDLite just took approximately two seconds. The two models performed well on both heights for the lowest distance, but then highly decreased their recall and confidence values. Although the SSDLite model could detect more individuals on the furtherer distances, it also started identifying other objects as person (i.e., FP). The following tests are performed in a scenario closer to a real situation where a quadcopter simulates the approach to the yard of a house from different heights and distances. With the camera of the DJI Phantom 4 and the SSD model, the person was not detected above 17.5 m. On the lower heights, from 2.5–7.5 m, the person was out of the camera’s range. We could conclude that for this model and camera, the best height is 10 m, which resulted in a recall of over 90%, consisting on 22 TP out of 24 people. In the same circumstances, the SSDLite found more people, while having lower confidence values and identifying other objects as if they were a person on the heights above 12.5 m, lowering the precision. In the cases where we tested the heights of 5 and 10 m, this model got 100% recall. According to the results of the second test scenario, the low-cost alternative, implemented for the third test scenario, was tested for heights up to 10 m. This height seemed to be the best point for higher recall, confidence, and no FP. Then, similarly to the previous tests, the SSD achieved lower recalls than the SSDLite model but higher confidence values. This means that when SSD detects a person, it is more likely to be a real person than a person identified by the SSDLite. However, the first model just got a maximum of ~33% recall for the 5 m height while the last model got recalls between ~59% and ~76%. In this test scenario, thanks to the camera’s wider field of view, we could capture the person in more distances for the same height, whereas the camera of the Phantom 4 could only capture the body of the receiver in closer distances. In other way, the footages of the Phantom 4, generally had better confidence values for the same height and distance conditions.

The fourth test scenario included different day conditions, from morning to night. The captures for the nighttime could not detect the person in any case, because of the dark images. For the afternoon conditions, the precision and recall of both algorithms was superior than in the morning. For instance, SSDLite presented recall values from ~78%–100% in the afternoon, compared to ~17%–60% in the morning. The precision is also slightly lower in the morning—33%–100% compared to 78%–100% in the afternoon. These results can be explained by the brighter sun light in the morning when compared to the afternoon.

It was considered, for the test, a confidence threshold of 0.5 (50%). The fifth and last test was made to compare this value to other possible thresholds—0.10–0.90 with 0.10 steps. The precision and recall values for the different thresholds were represented in a Precision–Recall curve, and demonstrated that the best thresholds are near the 0.50—for higher thresholds the precision increases but the recall drops a lot; for lower thresholds the precision decreases, which can be a problem for this solution: non-person objects should not be treated as real persons.

Summing up the results of every test scenario, both models presented patterns for the average time, precision, recall, and confidence. The time elapsed for processing the images with the SSD takes more than twice the time than with the SSDLite. Also, it can reach higher recall and confidence values, while it has lower precision and performance for the longer horizontal distances and higher heights. In these cases, the SSDLite performs better, finding almost all the persons while detecting other objects as persons and having low confidence values. Lastly, we can deduce that the approaching procedure should be done in two different steps. In the first step, at higher altitudes, the SSDLite is the proper algorithm to be used. With lower processing time and better detection range, it can detect the possible receivers present in the field of view. Then, the vehicle can get closer to the more confident predictions and proceed to the second step. For better precision and confidence values, it should be used the SSD model. Despite the higher processing time, a high confidence value with the SSD is a strong probability of a real person being detected.

Based on these results, there are significant illations. Relatively to the camera, the detection is highly dependent on its distance to the receiver and on the quality of its sensor. On the other hand, the performance of the tested deep-learning algorithms in low computing devices such as Raspberry Pi 3, do not bottleneck the execution of the proposed solution. This allows creation of a considerably fast detection cycle to find the possible receptor around the arriving location (less than three seconds for image acquisition and processing). The detection process executed over the pictures captured with the Pi Camera presented good results in the most probable approximation scenario. This scenario is defined with the drone being up to 10 m apart from the receiver, regarding the height, and up to 25 m apart from the receiver, regarding the horizontal distance.

Besides the brief analysis of the literature and tools related to the topic, this work contributes with a solution for a problem in one of the trendiest new generation services: autonomous deliveries with UASs. This problem is related to the dependency on the GPS for the landing process, after arriving at the target location. We analyzed an approach that allows definition of a dynamic landing point in the surroundings of the arrival coordinates according to the position of the receiver. In this case, it is required that the receiver be facing the drone and standing on a safe area. This strategy allows avoidance of landing problems created by low GPS accuracy or changes in the configuration of the scenery. For instance, this means that the drone will not land on a swimming pool, collide with a tree, or destroy its propellers against a new garden statuette. Therefore, we propose the architecture of a system that uses CV as the cornerstone to detect a human from an aerial perspective through the drone’s camera. This architecture was represented by a functional prototype that underwent five different test scenarios. The analysis of these results confirmed that it is possible to create a system to define a safe landing position in real time through cost-effective equipment (i.e., microcomputer with low computing power and low-quality camera). Moreover, since the processing device can be the flight controller itself, it is possible to implement this low-cost system without additional equipment besides the drone and its camera. The success of the tests also show that the human receiver is enough to define a landing point with no need for a specific visual marker.

As future work, we pretend to develop more tests in different conditions, for example during the night. Although the Federal Aviation Administration (FAA) does not allow UAV flight during the night, this step intend to validate and extend the functional robustness of this solution in different light conditions. The validation of the detection system considering the absence of light confirms its success in different scenarios that are not considered in this article (e.g., indoor deliveries, cave rescuing missions). The results regarding nighttime can also strengthen the detection behavior of our solution during daytime scenarios with decreased luminosity (e.g., cloudy days). Also, thanks to the use of a NoIR camera, our solution would not require another sensor to acquire imagery in low-luminosity scenarios. Instead it requires an infrared illumination module. We should also improve what we developed in this work and implement the complete delivery process. The first task should be to increase the flexibility of the detection system that currently is static. This means that if the drone arrives at the location and do not find a human, it should rotate its body or camera to scan the area around. If after a predefined number of attempts the vehicle does not detect a person, it should go back to the starting point. The next step of the delivery service is the acquisition of authorization from the potential package receiver, through facial recognition. The final phase consists of the interaction of the receiver with the drone, through movement patterns, detected with CV tools as well. Regarding the performance of the human detection system, the creation of a bigger dataset is one of the priorities, to create a more accurate model. This dataset should include, from different perspectives, a wider diversity of human morphology and poses, as much as other objects that were considered FP. To decrease the image processing time and increase the accuracy in the approaching step of the delivery, a cloud-based computing solution should be considered. This solution, allied to a drone connected to a 4G or 5G mobile network, should also be studied and tested in opposition to the DNN included in its Processing Module.

Finally, some considerations based on this study about remote sensing through UAS, focusing on autonomous landing for package delivery. We consider that the future of authenticated autonomous delivery passes by freeing the recipient from any visual markers representing the correct position. The authentication should be performed with ubiquitous devices or CV-based technologies such as facial recognition. The estimation of the landing location should be dynamic, since the environment around the predefined geolocation coordinates can change over time. We expect that in the future, an autonomous delivery UAS should be able to define the final landing spot in a safe area, near the authenticated recipient.

## Figures and Tables

**Table 1:** Summary of comparisons between the proposed method and previous studies.

Reference	Marker	Landing Approach	Space	Detection Target	Description	Strength	Weakness
[25]	Yes	Fixed	Outdoor	IR—LOCK	Using an Infrared Camera Sensor, detects the infrared emitter built-in in the landing platform	The IR-LOCK sensor works well on any lightning condition	Requires a landing platform equipped with infrared emitters
[27]	Yes	Fixed	Indoor	ArUco Marker	The solution lands a low-cost AR.Drone 2.0 on an indoor landing platform	Uses one camera to estimate the marker’s position and another to adjust the vehicle to land perfectly on the center of the marker	Requires a marker on the landing place. The processing is done by an external computer
[28]	Yes	Moving	Outdoor	AprilTag	The drone lands on a moving car by using a marker on its rooftop	This solution could land a drone on a car driving at 50 km/h	Requires a marker on the landing site. The car needs a smartphone to transmit its GPS location to the drone
[30]	Yes	Fixed	Outdoor	Custom Marker	Uses photo reconstruction methods on pictures captured by a cost-effective and low spatial resolution camera, to enhance them and detect the markers, using deep learning	The marker detection works with a low spatial resolution camera	Requires a marker on the landing platform
[31]	Yes	Moving	Outdoor	Color Filter	Detects the landing platform, in movement, to land on its top	Alternative and simplified approach to identify red contours on the images	Requires a marker on the landing place
[32]	Yes	Moving	Indoor	Image-Based Visual Servoing	Lands a UAV on a marker, using Image Visual Servoing	The vehicle can be controlled via three different modes: Patrol, Tracking, and Landing	The captures are transmitted and processed on an external computer via a 1.2 GHz video link
[33]	Yes	Fixed	Outdoor	Custom Marker	Detects the marker in real time	The detection Works on both day light and night daytimes	Requires a specific marker on the landing site
[34]	Yes	Fixed	Indoor	ALVAR AR Marker	System that lands an AR. Drone 2.0 autonomously	Uses a low-cost UAV. The commands are sent via a WiFi link	The marker’s detection processing is made on an external device
[35]	Yes	Moving	Indoor	Multiple Simple AprilTag	Detects a landing platform in movement and lands the UAV on it	The vehicle handles all the processing	The smaller markers inside the larger one introduces noise in its recognition
[36]	No	Moving	Outdoor	Cattle Detection	Detects animals on a farm, using deep learning models	Detects animals without the need for a marker	The processing is not made at runtime
[37]	Yes	Fixed	Outdoor or Indoor	ArUco Marker	Detects and identifies markers using a low-cost camera	The processing is done onboard	Requires a marker to identify the landing site
[38]	Yes	Fixed	Outdoor	Custom Marker	Uses a CNN lightDenseYOLO model to predict the marker’s location	Detects the marker at long distances	Requires a dedicated system to detect the marker in real time
This work	No	Fixed	Outdoor	People Detection	Defines the landing site dynamically based on a person detection	Dynamic landing place, without using specific markers	Success depends on the human factor

**Table 2:** Test case scenarios and respective goals.

Test Case	Goals	Output	Scenario Conditions	Camera Used
1	Test and compare the speed and accuracy of algorithms	Speed, precision, recall, confidence	Controlled, stationary	iPhone 6S Plus
2	Assess the performance on a real case with commercial vehicle	Balance between precision, recall, height, and distance	Built-in	DJI Phantom 4 Camera
3	Compare the difference between low-cost camera with a mid-range one	Balance between precision, recall, height, and distance	Fixed to a UAV	Raspberry Pi NoIR Camera V2 fixed to DJI Phantom 4
4	Evaluate the algorithms’ accuracy under different daytimes	Balance between precision, recall, height, and distance	Fixed to a UAV	Raspberry Pi NoIR Camera V2 fixed to DJI Phantom 4
5	Appraise the ratio between precision and recall, using different threshold values	Precision–Recall curve—best threshold, precision, and recall values	Fixed to a UAV	Raspberry Pi NoIR Camera V2 fixed to DJI Phantom 4

**Table 3:** Compilation of the results of the test for the first case scenario on the Floor 1 (4.5 m).

Dist.	Model	Samples	Total	TP	FN	FP	Avg. Conf.	Precision	Recall	Avg. Time
5 m	SSD-MN-V2	24	48	34	14	0	0.7762	1	0.7083	4.6697
10 m		45	83	12	71	0	0.6856	1	0.1446	4.6433
15 m		46	90	2	88	1	0.5678	0.6667	0.0222	4.6482
30 m		130	255	1	254	1	0. 5477	0.5000	0.0039	4.6064
5 m	SSDLite-MN-V2	24	48	45	3	11	0.6715	0.8036	0.9375	1.9793
10 m		45	83	24	59	20	0.6033	0.5455	0.2892	1.9728
15 m		46	90	11	79	17	0.5502	0.3929	0.1222	1.9681
30 m		130	255	30	225	36	0.6121	0.4545	0.1176	1.9659

**Table 4:** Compilation of the results of the test for the first case scenario on the Floor 2 (10 m).

Dist.	Model	Samples	Total	TP	FN	FP	Avg. Conf.	Precision	Recall	Avg. Time
5 m	SSD-MN-V2	67	134	79	55	0	0.7025	1	0.5896	4.6707
10 m		40	90	7	83	2	0.6289	0.7778	0.0778	4.6390
15 m		48	94	0	94	1	-	0.0000	0.0000	4.6759
30 m		72	136	6	130	1	0.7831	0.8571	0.0441	4.7639
5 m	SSDLite-MN-V2	67	134	103	31	0	0.6266	1	0.7687	1.9715
10 m		40	90	5	85	20	0.6408	0.2000	0.0556	1.9705
15 m		48	94	0	94	18	-	0.0000	0.0000	1.9623
30 m		72	136	17	119	11	0.5703	0.6071	0.1250	1.9752

**Table 5:** Second test scenario results for the SSD and SSDLite algorithms.

Model	Height	Total	TP	FN	FP	Precision	Recall
**SSD-MN-V2**	2.5	6	6	0	0	1	1
	5	15	11	4	0	1	0.7333
	7.5	15	12	3	0	1	0.8000
	10	24	22	2	0	1	0.9167
	12.5	27	19	8	0	1	0.7037
	15	27	11	16	0	1	0.4074
	17.5	27	5	22	0	1	0.1852
	20	27	0	27	0	-	0.0000
	22.5	24	0	24	0	-	0.0000
	25	24	0	24	0	-	0.0000
**SSDLite-MN-V2**	2.5	9	8	1	0	1	0.8889
	5	15	15	0	0	1	1
	7.5	21	18	3	0	1	0.8571
	10	27	27	0	0	1	1
	12.5	27	25	2	0	1	0.9259
	15	27	25	2	19	0.5682	0.9259
	17.5	27	27	0	26	0.5094	1
	20	27	26	1	23	0.5306	0.9630
	22.5	24	22	2	22	0.5000	0.9167
	25	24	17	7	15	0.5313	0.7083

**Table 6:** True positives comparison for SSD-MN-V2 on the second test scenario.

		Distance (m)									
		2.5	5	7.5	10	12.5	15	17.5	20	22.5	25
**Height (m)**	25	OR	OR	0	0	0	0	0	0	0	0
	22.5	OR	OR	0	0	0	0	0	0	0	0
	20	OR	0	0	0	0	0	0	0	0	0
	17.5	OR	0	0	0	2	1	0	0	2	0
	15	OR	1	1	2	1	1	1	1	0	3
	12.5	OR	1	2	3	1	3	2	2	3	2
	10	OR	3	2	3	3	3	2	3	3	IP
	7.5	IP	3	2	3	2	2	IP	OR	OR	OR
	5	3	3	2	2	1	OR	OR	OR	OR	OR
	2.5	3	3	IP	OR	OR	OR	OR	OR	OR	OR
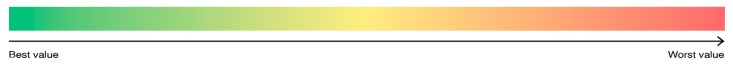											

**Table 7:** Average confidence comparison for SSD-MN-V2 on the second test scenario.

		Distance (m)									
		2.5	5	7.5	10	12.5	15	17.5	20	22.5	25
**Height (m**)	25										
	22.5										
	20										
	17.5					0.5950	0.5144			0.7582	
	15		0.8217	0.5463	0.6880	0.7743	0.5503	0.5841	0.6836		0.7274
	12.5		0.7306	0.6529	0.7727	0.5966	0.6739	0.7620	0.7176	0.8601	0.7736
	10		0.7302	0.6296	0.8109	0.8312	0.6558	0.6537	0.8106	0.8161	
	7.5		0.8971	0.7148	0.8620	0.8675	0.8856				
	5	0.9540	0.8159	0.6920	0.6787	0.5128					
	2.5	0.9758	0.7180								
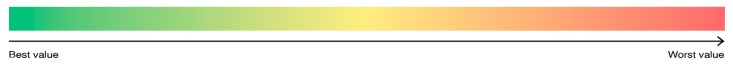											

**Table 8:** True positives comparison for the SSDLite-MN-V2 on the second test scenario.

		Distance (m)									
		2.5	5	7.5	10	12.5	15	17.5	20	22.5	25
**Height (m)**	25	OR	OR	3	2	2	3	2	2	1	2
	22.5	OR	OR	3	3	2	2	3	3	3	3
	20	OR	3	3	2	3	3	3	3	3	3
	17.5	OR	3	3	3	3	3	3	3	3	3
	15	OR	2	3	3	3	3	3	2	3	3
	12.5	OR	2	3	3	3	3	3	3	3	2
	10	OR	3	3	3	3	3	3	3	3	3
	7.5	2	3	3	3	3	3	1	OR	OR	OR
	5	3	3	3	3	3	OR	OR	OR	OR	OR
	2.5	3	3	2	OR	OR	OR	OR	OR	OR	OR
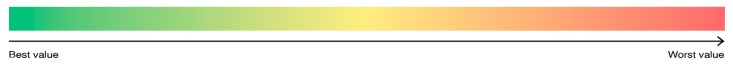											

**Table 9:** Average confidence comparison for the SSDLite-MN-V2 on the second test scenario.

		Distance (m)									
		2.5	5	7.5	10	12.5	15	17.5	20	22.5	25
**Height (m)**	25			0.5360	0.5454	0.5472	0.5366	0.5400	0.5438	0.5335	0.5566
	22.5			0.5594	0.5510	0.5293	0.5236	0.5888	0.5955	0.6155	0.6080
	20		0.6432	0.6016	0.5545	0.5527	0.6178	0.6360	0.6195	0.5832	0.5769
	17.5		0.5771	0.6034	0.6081	0.6786	0.6952	0.7448	0.6821	0.6832	0.6454
	15		0.5185	0.5467	0.6598	0.6193	0.7228	0.6952	0.7423	0.6616	0.6337
	12.5		0.6339	0.6622	0.6268	0.6552	0.7179	0.6942	0.6482	0.7145	0.6081
	10		0.6659	0.6335	0.7341	0.7260	0.6679	0.6507	0.7150	0.7377	0.6844
	7.5	0.5112	0.6737	0.6519	0.6797	0.6981	0.6943	0.5130			
	5	0.7932	0.7657	0.6321	0.6831	0.5634					
	2.5	0.8456	0.6474	0.5855							
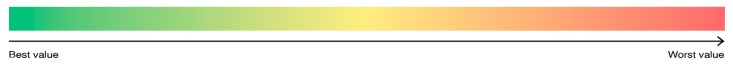											

**Table 10:** Third test scenario results for the SSD and SSDLite algorithms.

Model	Height	Total	TP	FN	FP	Precision	Recall
SSD-MN-V2	2.5	30	8	22	0	1	0.2667
	5	27	9	18	0	1	0.3333
	7.5	24	3	21	0	1	0.1250
	10	18	2	16	0	1	0.1111
SSDLite-MN-V2	2.5	30	21	9	0	1	0.7000
	5	27	16	11	0	1	0.5926
	7.5	24	16	8	0	1	0.6667
	10	21	16	5	2	0.8889	0.7619

**Table 11:** True positives comparison for the SSD-MN-V2 on the third test scenario.

		Distance (m)									
		2.5	5	7.5	10	12.5	15	17.5	20	22.5	25
**Height (m)**	10	OR	OR	OR	IP	0	1	0	1	0	0
	7.5	OR	IP	1	0	1	1	0	0	0	0
	5	OR	3	3	1	1	1	0	0	0	0
	2.5	3	3	2	0	0	0	0	0	0	0
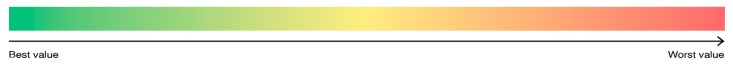											

**Table 12:** Average confidence comparison for the SSD-MN-V2 on the third test scenario.

		Distance (m)									
		2.5	5	7.5	10	12.5	15	17.5	20	22.5	25
**Height (m)**	10						0.7909		0.7428		
	7.5			0.5212		0.7474	0.5461				
	5		0.8426	0.7331	0.8288	0.6024	0.5006				
	2.5	0.8689	0.8768	0.6764							
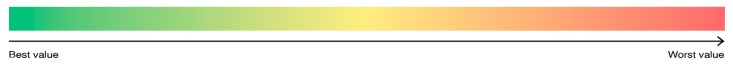											

**Table 13:** True positives comparison for SSDLite-MN-V2 on the third test scenario.

		Distance (m)									
		2.5	5	7.5	10	12.5	15	17.5	20	22.5	25
**Height (m)**	10	OR	OR	OR	1	3	3	2	3	3	1
	7.5	OR	IP	2	3	3	3	3	2	0	0
	5	OR	3	3	3	3	3	0	1	0	0
	2.5	3	3	2	2	3	3	1	2	1	1
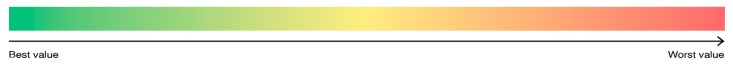											

**Table 14:** Average confidence comparison for SSDLite-MN-V2 on the third test scenario.

		Distance (m)									
		2.5	5	7.5	10	12.5	15	17.5	20	22.5	25
**Height (m)**	10				0.5253	0.5588	0.6016	0.5627	0.6171	0.5785	0.5154
	7.5			0.5630	0.6765	0.6232	0.5853	0.5848	0.5720		
	5		0.7616	0.6807	0.6383	0.6393	0.5611		0.5257		
	2.5	0.7639	0.7483	0.6734	0.6040	0.5774	0.6257	0.6406	0.5131	0.5704	0.5302
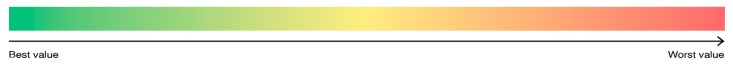											

**Table 15:** Fourth test scenario results for the SSD and SSDLite algorithms.

Model	Daytime	Height	Total	TP	FN	FP	Precision	Recall
SSD-MN-V2	Morning	2.5	18	8	10	0	1	0.4444
		5	15	1	14	0	1	0.0667
		7.5	15	0	0	0	-	0
		10	6	0	6	0	-	0
	Afternoon	2.5	18	8	10	0	1	0.4444
		5	15	9	6	0	1	0.6000
		7.5	12	3	9	0	1	0.2500
		10	6	1	5	0	-	0.1667
SSDLite-MN-V2	Morning	2.5	18	7	11	0	1	0.3889
		5	15	9	6	1	0.9000	0.6000
		7.5	15	5	10	3	0.6250	0.3333
		10	6	1	5	2	0.3333	0.1667
	Afternoon	2.5	18	16	2	0	1	0.8889
		5	15	15	0	0	1	1
		7.5	12	11	1	0	1	0.9167
		10	9	7	2	2	0.7778	0.7778

**Table 16:** True positives comparison for SSD-MN-V2 on the fourth test scenario, during morning.

		Distance (m)					
		2.5	5	7.5	10	12.5	15
**Height (m)**	10	OR	OR	OR	IP	0	0
	7.5	OR	IP	0	0	0	0
	5	OR	1	0	0	0	0
	2.5	3	3	2	0	0	0
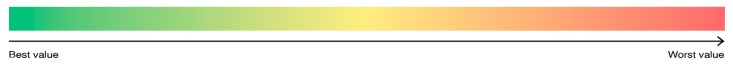							

**Table 17:** Average confidence comparison for SSD-MN-V2 on the fourth test scenario, during morning.

		Distance (m)					
		2.5	5	7.5	10	12.5	15
**Height (m)**	10						
	7.5						
	5		0,5978				
	2.5	0,9601	0,7402	0,6079			
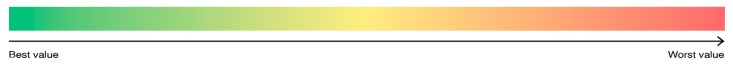							

**Table 18:** True positives comparison for SSDLite-MN-V2 on the fourth test scenario, during morning.

		Distance (m)					
		2.5	5	7.5	10	12.5	15
**Height (m)**	10	OR	OR	OR	IP	0	1
	7.5	OR	2	1	1	0	1
	5	OR	3	0	1	2	3
	2.5	3	3	1	0	0	0
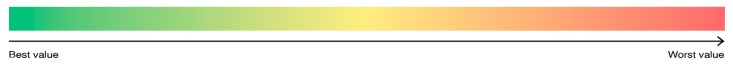							

**Table 19:** Average confidence comparison for SSDLite-MN-V2 on the fourth test scenario, during morning.

		Distance (m)					
		2.5	5	7.5	10	12.5	15
**Height (m)**	10						0,5370
	7.5		0,5554	0,5348	0,5661		0,6328
	5		0,5872		0,5596	0,5662	0,5301
	2.5	0,6790	0,6633	0,6016			
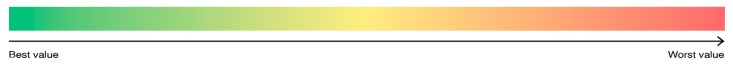							

**Table 20:** True positives comparison for the SSD-MN-V2 on the fourth test scenario, during afternoon.

		Distance (m)					
		2.5	5	7.5	10	12.5	15
**Height (m)**	10	OR	OR	OR	IP	0	1
	7.5	OR	IP	1	0	1	1
	5	OR	3	3	1	1	1
	2.5	3	3	2	0	0	0
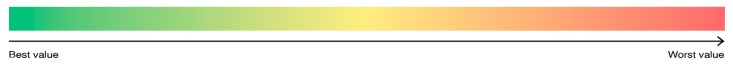							

**Table 21:** Average confidence comparison for the SSD-MN-V2 on the fourth test scenario, during afternoon.

		Distance (m)					
		2.5	5	7.5	10	12.5	15
**Height (m)**	10						0.7909
	7.5			0.5212		0.7474	0.5461
	5		0.8426	0.7331	0.8288	0.6024	0.5006
	2.5	0.8689	0.8768	0.6764			
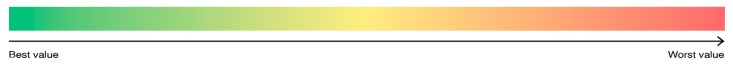							

**Table 22:** True positives comparison for SSDLite-MN-V2 on the fourth test scenario, during afternoon.

		Distance (m)					
		2.5	5	7.5	10	12.5	15
**Height (m)**	10	OR	OR	OR	1	3	3
	7.5	OR	IP	2	3	3	3
	5	OR	3	3	3	3	3
	2.5	3	3	2	2	3	3
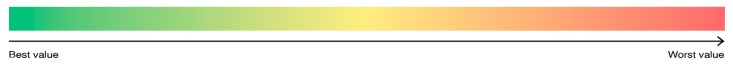							

**Table 23:** Average confidence comparison for SSDLite-MN-V2 on the fourth test scenario, during afternoon.

		Distance (m)					
		2.5	5	7.5	10	12.5	15
**Height (m)**	10				0.5253	0.5588	0.6016
	7.5			0.5630	0.6765	0.6232	0.5853
	5		0.7616	0.6807	0.6383	0.6393	0.5611
	2.5	0.7639	0.7483	0.6734	0.6040	0.5774	0.6257
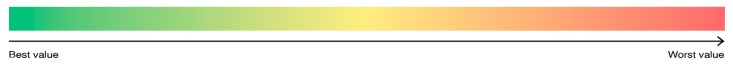							

**Table 24:** Fifth test scenario results, including the precision for each confidence threshold and the correspondent precision and recall values.

Threshold	TP	FN	FP	Precision	Recall	F0.5-Score
0.10	105	0	2283	0.04	1.00	0.0495
0.20	105	0	790	0.12	1.00	0.1456
0.30	102	3	114	0.47	0.97	0.5240
0.40	89	16	6	0.94	0.85	0.9205
0.50	69	36	2	0.97	0.66	0.8867
0.60	38	67	0	1.00	0.36	0.7377
0.70	14	91	0	1.00	0.13	0.4276
0.80	0	105	0	-	0.00	-
0.90	0	105	0	-	0.00	-
